# Dark chocolate supplementation reduces the oxygen cost of moderate intensity cycling

**DOI:** 10.1186/s12970-015-0106-7

**Published:** 2015-12-15

**Authors:** Rishikesh Kankesh Patel, James Brouner, Owen Spendiff

**Affiliations:** School of Life Sciences, Kingston University, Kingston upon Thames, KT1 2EE UK

**Keywords:** Gas exchange threshold, Flavanols, High-intensity, Time trial

## Abstract

**Background:**

Dark chocolate (DC) is abundant in flavanols which have been reported to increase the bioavailability and bioactivity of nitric oxide (NO). Increasing NO bioavailability has often demonstrated reduced oxygen cost and performance enhancement during submaximal exercise.

**Methods:**

Nine moderately-trained male participants volunteered to undertake baseline (BL) measurements that comprised a cycle $\overset{.}{V}{O}_{2 max}$ test followed by cycling at 80 % of their established gas exchange threshold (GET) for 20-min and then immediately followed by a two-minute time-trial (TT). Using a randomised crossover design participants performed two further trials, two weeks apart, with either 40 g of DC or white chocolate (WC) being consumed daily. Oxygen consumption, RER, heart rate and blood lactate (BLa) were measured during each trial.

**Results:**

DC consumption increased GET and TT performance compared to both BL and WC (*P* < 0.05). DC consumption increased $\overset{.}{V}{O}_{2 max}$ by 6 % compared to BL (*P* < 0.05), but did not reach statistical significance compared to WC. There were no differences in the moderate-intensity cycling for $\overset{.}{V}{O}_2$_,_ RER, BLa and heart rate between conditions, although, $\overset{.}{V}{O}_2$ and RER exhibited consistently lower trends following DC consumption compared to BL and WC, these did not reach statistical significance.

**Conclusion:**

Chronic supplementation with DC resulted in a higher GET and enhanced TT performance. Consequently, ingestion of DC reduced the oxygen cost of moderate intensity exercise and may be an effective ergogenic aid for short-duration moderate intensity exercise.

## Background

Dietary supplementation with sodium nitrate or nitrate rich beetroot juice has become increasingly popular and has consistently been shown to reduce oxygen demands during submaximal exercise and resting blood pressure (BP) [1, 2]. The physiological mechanisms underpinning these enhancements in contractile efficiency have been attributed to the breakdown of nitrate to the substantial elevation of circulating nitrite, which is subsequently converted into the bioactive nitric oxide (NO), that may potentially increase vasodilation, glucose uptake and regulate muscular contraction [3–7].

Although supplementation with beetroot juice has become popular as an ergogenic aid, it is renowned for its poor palatability and lack of energy provision. Recent studies reported similar vascular improvements in NO levels from consumption of dark chocolate (DC) [8, 9]. This is of great interest given the popularity of DC often associated with food categories of high palatability and indulgence. Dark chocolate mechanistically unique to other dietary nitrate supplements augments NO production through endothelium-dependent influences [10, 11]. The abundant amount of (-)-epicatechin found within DC signals release of vasoactive components from the endothelial cells increasing the bioavailability of NO [12, 13]. The increased bioavailability and activity of NO have been demonstrated to statisitically increase flow-mediated dilation in healthy patients (46 g/day for fifteen days) [14], hypertensive patients (100 g/day for fifteen days) [15], pre-hypertensive patients (30 g/day for fifteen days) [13], and stage 1 hypertension patients (6.3 g/day for eighteen weeks) [16].

To date, the research surrounding DC has predominantly focused on its beneficial influences on cardiovascular health and has received limited focus towards the effects on exercise performance. Berry et al. [17] demonstrated attenuation in exercise-induced blood pressure through consumption of flavanol rich cocoa within overweight participants, demonstrating DC may decrease cardiovascular risk and enhance the cardiovascular benefits of moderate intensity exercise in at-risk individuals. Allgrove et al. [18] reported consumption of DC (40 g) for two weeks was associated with reduced oxidative-stress markers following prolonged exhaustive exercise and increased mobilization of free fatty acids after exercise. Allgrove et al. [18] and Berry et al. [17] also suggested that increased NO levels resulted in lower RER and improvements in moderate intensity.

To date, no studies have addressed the link between the reported increases in NO and nitrate levels resulting from consumption of DC and for its potential to lower oxygen demand during moderate intensity exercise and enhance performance during high-intensity exercise. Therefore, the purpose of this study was to examine whether commercially available DC, will elicit an improvement in gas exchange threshold (GET), lower oxygen consumption during moderate intensity cycling (80 % GET) and improve performance in a time trial in comparison to white chocolate.

## Materials and methods

### Participants

Following Institutional approval nine moderately trained males (mean ± SD; Age 21 ± 1 years, body mass 76.0 ± 9.3 kg, stature 177 ± 9.4 cm and $\overset{.}{V}{O}_{2 max}$ 41.89 ± 5.4 ml/kg/min) volunteered to participate and provided written informed consent to participate in the study. All procedures and conduct met with Declaration of Helsinki.

### Dietary control

Participants were instructed to maintain their normal diet and refrain from alcohol, vitamin supplements, anti-inflammatory products and from consumption of milk two hours before and after consumption of either supplementation, to increase absorption over the period of intervention. Participants were also provided with a dietary list of prohibitory foods high in nitrate with an appropriate low nitrate replacement. To prevent an increase surplus in daily caloric energy intake, participants replaced a snack meal or dessert with similar caloric values for DC/WC and were instructed not reduce their fruit and vegetable consumption. The DC and WC were best matched for calorific content (total energy provision over 14 days was 12887 kJ & 12945 kJ for DC and WC respectively) and no other supplementation was allowed throughout the intervention including consuming any other chocolate. In the 24 h preceding the first exercise test, participants were asked to record their dietary intake, detailing all foods consumed, and this diet was replicated in the 24 h preceding subsequent tests. Participants were instructed to avoid any strenuous activity in the 24 h preceding each testing session, to refrain from caffeine for six hours and from alcohol for 24 h before each testing session. During the 2-week washout period between interventions, participants were not required to adhere to any specific dietary regime, but to avoid chocolate.

### Experimental design

**Fig. 1:**
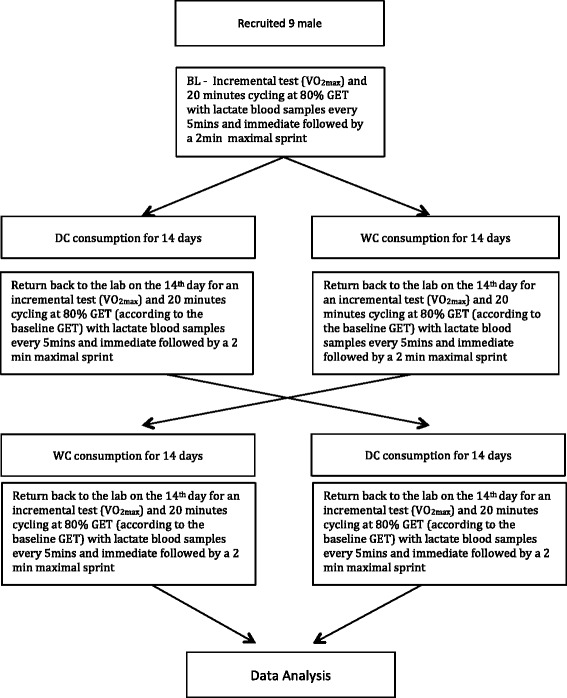
Schematic illustration of the exercise test protocol

To examine whether dark chocolate consumption affects $\overset{.}{V}{O}_{2 max}$ (ml/kg/min), blood pressure (BP) (mmHg), oxygen cost (ml/kg/min), and lactate levels (mmol/L) during a 20-min cycle test at 80 % GET and all out sprint performance (m), a randomised crossover design was used with participants blinded to the aims of the study. Baseline tests (BL) were used to accustom participants to testing protocols (Fig. 1), establish GET for the entirety of the protocol, and for participants to be randomly assigned to either a daily intake of DC (40 g, DOVE®, Dark Chocolate, Mars, Incorporated, Hackettstown, NJ) or white chocolate (WC) (40 g of Milkybar®) for fourteen days, followed by a seven day wash out period, then switched to the alternative treatment. The commercially available dark chocolate was chosen as it was previously reported to be rich in (-)-epicatechin [14]. Fourteen days of supplementation was used as Sudarma et al. [13] reported increases in NO following DC consumption of this quantity an duration.

### Equipment and procedures

All three laboratory visits were performed at the same time of day on different days and performed in a standardised order (Fig. 1).

### Resting measures

Stature was measured to the nearest 0.1 cm using stadiometer and body mass was measured to the nearest 0.05 kg using an electronic scales (Seca, Vogel & Halke, Germany). Participants were rested supine for five minutes before BP measurements were taken using a Maruman digital automatic BP monitor (Maruman Co. Ltd., Tokyo, Japan). On each occasion, three measurements were taken, with a 60s rest between each measurement and the mean value reported.

### Incremental test $\left(\overset{.}{V}{O}_{2 max}\right)$

All tests were performed on an electromagnetically braked cycle ergometer (Velotron Cycles, Racermate Inc., Seattle, Washington, USA). A ramp incremental test was used in accordance with Bailey et al. [1] procedures with the primary purpose to establish each participant GET. Participants cycled at a self-selected pedal rate between 70–90 rpm for 3-min ‘unloaded’ (0 W) warm up, after which the work rate was increased at a rate of 30 W/min until volitional exhaustion. Pedal rate, saddle height, and handle bar height configuration were recorded and reproduced in subsequent tests. Pulmonary gas exchange was continually collected throughout the incremental test (Oxycon Pro, VIASYS GmbH, Eric Jaeger, Hoechberg, Germany) and was averaged to fifteen seconds collection periods. $\overset{.}{V}{O}_{2 max}$ was recorded as the highest value achieved over a 15-s collection period.

### Moderate intensity cycle

Participants rested for 30 min after completing the incremental test, before commencing moderate cycling. To ensure consistency across conditions, the GET and corresponding power output for all three conditions (BL, DC, WC) was calculated from the BL $\overset{.}{V}{O}_{2 max}$ test. The GET from the baseline test was determined as the work rate corresponding at the precise cross over, at which V_E_/ $\overset{.}{V}{O}_2$ continually exceed V_E_/VCO_2_. A power output corresponding to 80 % of GET was used as the moderate cycling intensity for twenty minutes at a self-selected pedal cadence. Heart rate and pulmonary gas exchange were collected continually throughout the 20-min cycle and averaged to five-minute intervals. Capillary blood was collected from the index fingertip every five minutes and analysed for concentrations of lactate (Arkray Factory Inc, 1480 Oaza Kouji umeda, kounan-cho kouka gmn, Shiga, Japan).

### Distance time trial

On completion of the moderate intensity cycling, participants were instructed to cycle maximally for two minutes and the total distance (m) achieved was recorded.

### Statistical analysis

Data was analysed for normality using Shapiro Wilks. A two factor within-subject repeated measures ANOVA was used to analyse the differences over the 20 min of moderate intensity exercise (oxygen consumption, RER, lactate levels and heart rate) with Newman Keuls post-hoc tests used when differences were found. A paired t-test was used to measure the difference between all other dependent variables. Data was presented as mean ± standard deviation (SD), unless otherwise stated. Statistical significance was accepted when *P* ≤ 0.05.

## Results

### Maximal oxygen consumption

**Fig. 2:**
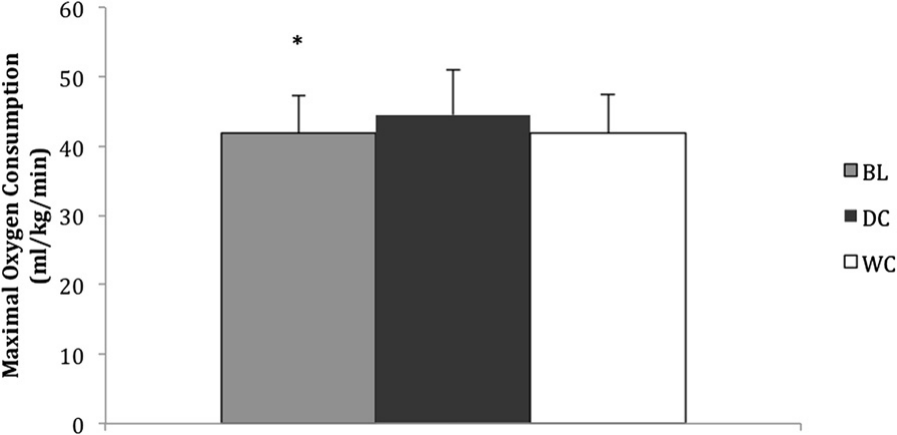
Maximal oxygen consumption (ml/kg/min) of participants at BL and after consumption of DC (*n* = 9) and WC (*n* = 9). *Denotes significant difference between DC (*P* ≤ 0.05)

Maximal oxygen consumption was 6 % higher following DC consumption (44.52 ± 6.43 ml/kg/min) compared to BL (41.89 ± 5.4 ml/kg/min, *P* = 0.037, 95 % CI = 5.05–0.21, Fig. 2) and also 6 % greater than that of WC (41.84 ± 5.6 ml/kg/min, *P* = 0.071, Fig. 2), but this did not reach statistical significance. There was no difference between maximal oxygen consumption following WC consumption compared to BL.

### Gas exchange threshold

**Fig. 3:**
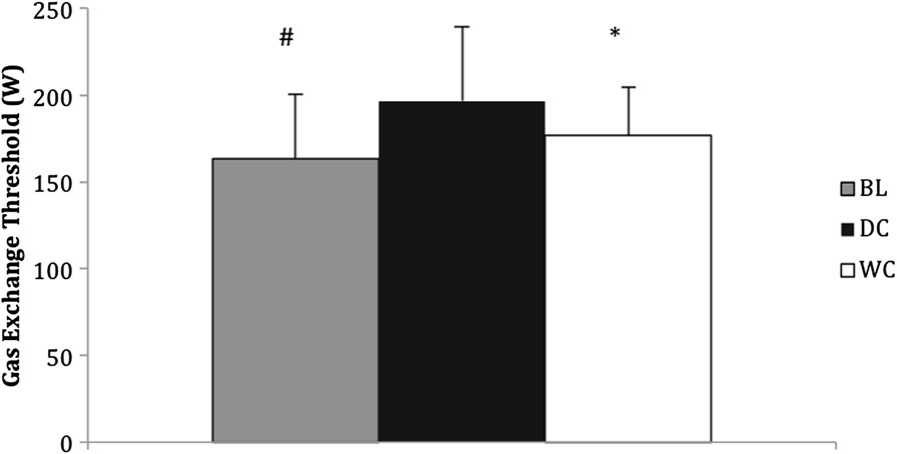
Gas exchange threshold (W) at BL and after consumption of DC (*n* = 9) and WC (*n* = 9). *Denotes significant difference between DC and BL (*P* < 0.05). # Denotes a significant difference between DC and WC (*P* < 0.05)

GET for both conditions was estimated from the $\overset{.}{V}{O}_{2 max}$ test undertaken for that condition post exercise. DC consumption (197 ± 43 W) significantly increased gas exchange threshold by 21 % compared to BL (163 ± 37 W, *P* = 0.007, 95 % CI = 54.73–11.93, Fig. 3) and was 11 % higher compared to WC (177 ± 28 W, *P* = 0.05, 95 % CI = 0.29–39.97, Fig. 3). Gas exchange threshold was not different following consumption of WC compared to BL (*P* = 0.225, Fig. 3).

### Oxygen consumption during the moderate intensity cycle

**Fig. 4:**
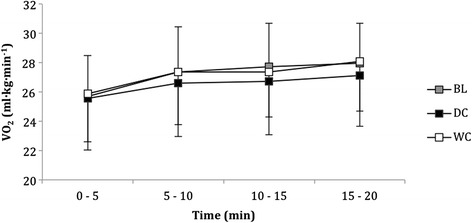
Oxygen consumption (ml/kg/min) of participants over twenty minutes during the moderate intensity cycle at BL and after consumption of DC (*n* = 9) and WC (*n* = 9)

All 20-min sub-maximal tests were undertaken at an intensity corresponding to 80 % of GET obtained in the BL trial. Oxygen consumption during the moderate intensity cycle was not different between conditions (*P* = 0.269, Eta = 0.151, Fig. 4). However, there were within-trial differences for all conditions that increased across time between 0–20 min (*P* < 0.001, Fig. 4).

### Respiratory exchange ratio during the moderate intensity cycle

**Fig. 5:**
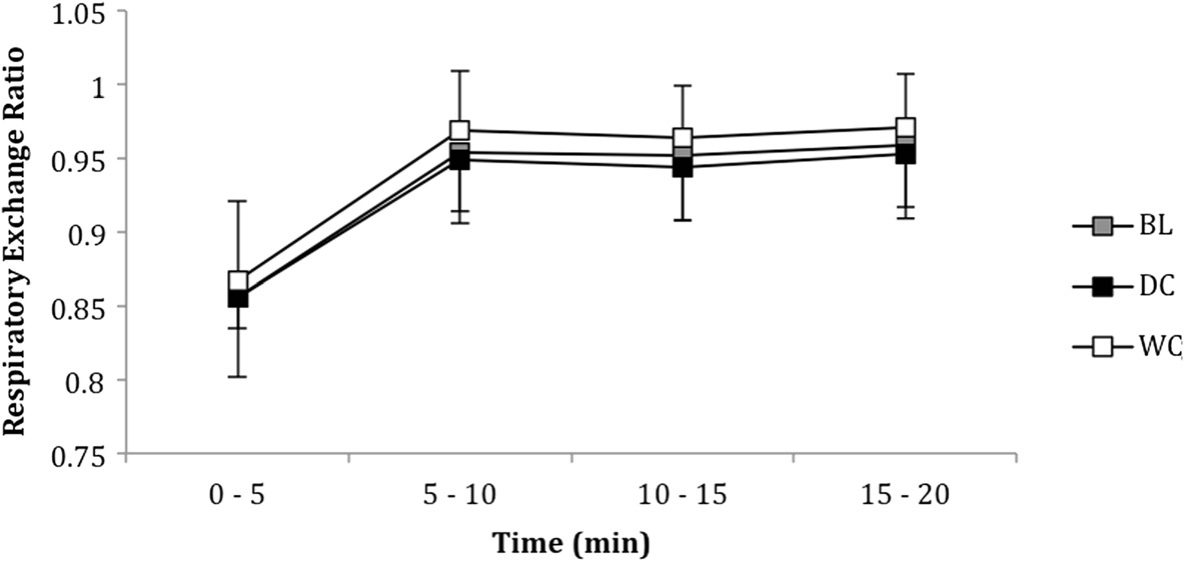
Respiratory exchange ratio of participants over twenty minutes during the moderate intensity cycle at BL and after consumption of DC (*n* = 9) and WC (*n* = 9)

Respiratory exchange ratio was not different between conditions (*P* = 0.488, Eta = 0.86 Fig. 5). However, there were within-trial differences for all conditions that increased across time between 0 to 20 min (*P* < 0.001, Fig. 5).

### Blood pressure

**Fig. 6:**
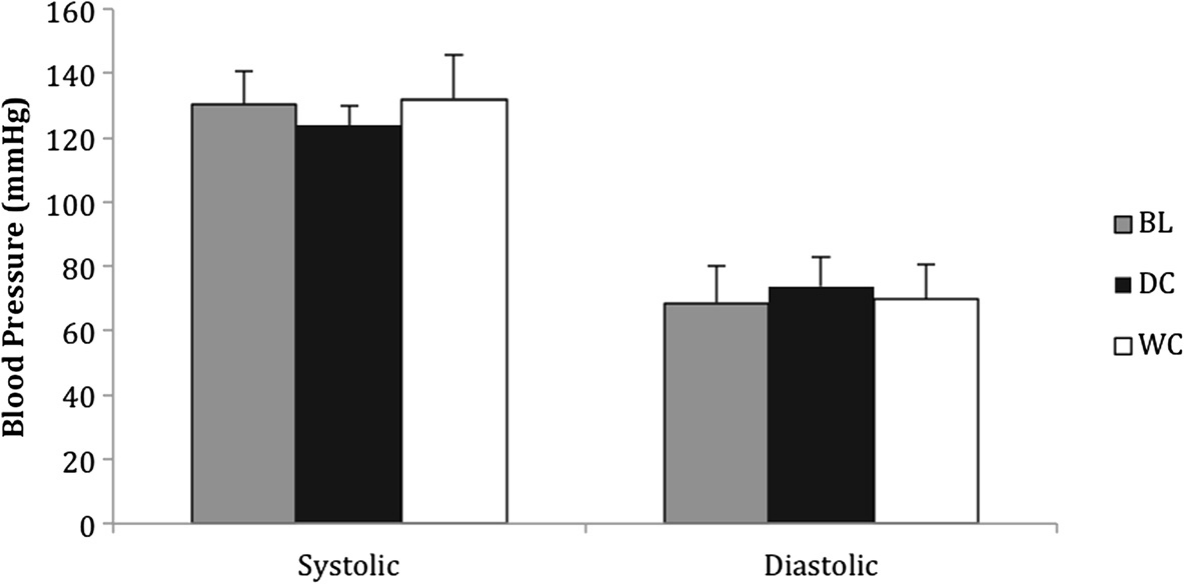
Systolic and diastolic blood pressure (mmHg) at BL and after consumption of DC (*n* = 9) and WC (*n* = 9)

Systolic BP was not different between conditions (*P* = 0.101, Fig. 6), DC resulted in a 6 % decrease in systolic blood compared to WC and 5 % decrease compared to BL; although these difference did not reach statistical significance. Diastolic BP was not different between conditions (*P* = 0.531, Fig. 6), although DC resulted in a 6 % increase in diastolic BP compared to WC and BL, but this did not reach statistical significance.

### Blood lactate during the moderate intensity cycle

**Fig. 7:**
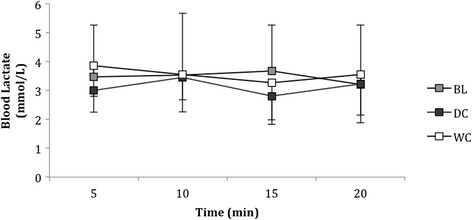
Blood lactate (mmol/L) of participants during the twenty minute moderate intensity cycle taken at 5 min, 10 min, 15 min and 20 min at BL and after consumption of DC and WC

Blood lactate was not different between conditions (*P* = 0.456, Eta = 0.093, Fig. 7). However, there were within-trial differences for all conditions across time that had no consistent identifiable trends (*P* = 0.479, Eta = 0.096, Fig. 7).

### Heart rate during the moderate intensity cycle

**Fig. 8:**
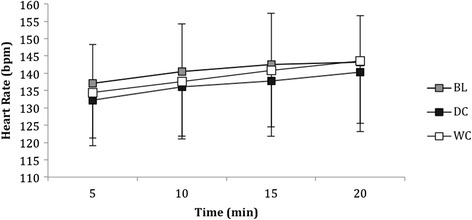
Heart rate (bpm) of participants during the twenty minute moderate intensity cycle taken at 5 min, 10 min, 15 min and 20 min at BL and after consumption of DC and WC

Heart rate was not different between conditions (*P* = 0.245, Eta = 0.161, Fig. 8). However, there were within-trial differences for all conditions that increased across time between 0–20 min (*P* = 0.001, Eta = 0.62, Fig. 8).

### Distance time trial

**Fig. 9:**
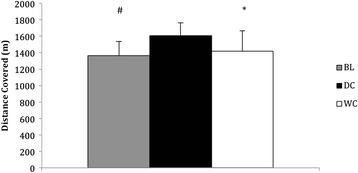
Total distanced cover (m) by participants during the two minute time trial at BL and after consumption of DC (*n* = 9) and WC (*n* = 9). *Denotes significant difference between DC and BL (*P* < 0.05). # Denotes a significant difference between DC and WC (*P* < 0.05)

DC consumption resulted in a greater total distance covered of 17 % (1606 ± 158 m, *P* = 0.001, 95 % CI = 312.76–165.01, Fig. 9) compared to BL (1367 ± 171 m) and a 13 % increase compared to WC (1419 ± 248 m, *P* = 0.003, 95 % CI = 291.21–82.11, Fig. 9). Total distance covered during the time trial was not different following consumption of WC compared to BL (*P* = 0.319, Fig. 9)

## Discussion

To date, this is the first study to investigate the effects of Dark Chocolate (DC) on $\overset{.}{V}{O}_{2 max}$, GET, oxygen demand during moderate intensity cycling, and a maximal performance time trial. The main finding was that regular consumption of DC daily (40 g) for fourteen days resulted in a significantly higher GET (21 and 11 %) and the total distance covered (17 and 13 %) during a maximal two minute maximal sprint compared to both baseline and WC conditions. Consumption of DC increased $\overset{.}{V}{O}_{2 max}$ compared to baseline (*P* = 0.037). However, WC also marginally increased $\overset{.}{V}{O}_{2 max}$ compared to baseline and this increase reduced the difference between WC and DC resulting in statistical significance not attaining the determined critical threshold (*P* = 0.071). There were no differences in the moderate intensity cycling in $\overset{.}{V}{O}_2$ RER, BLa, and heart rate between conditions. Furthermore there was no difference on systolic BP and diastolic BP between conditions.

While the specific mechanisms underlying this increase in GET and 2-min maximal sprint remains elusive it can be proposed that flavanol a subclass of DC is a particular compound of interest and may be responsible for eliciting the observed effects [3, 5, 19–21]. Dark Chocolate (DC), unique to other dietary nitrate supplements is hypothesised to mediate NO production through endothelium-dependent effects; related to flavanols’ ability to supress vascular arginase enzyme activity, thus increasing the production of NO [4, 13, 22, 23]. Dark chocolate’s vasodilatative properties can be attributed to the increase in levels of (-)-epicatechin, which are further converted to *0-*methylated metabolites that are potent inhibitors of NADPH oxidase. This suppreses oxygen free radical generation which increases the bioavailability of NO [10, 11]. Fisher et al. [24] and Sudarma et al. [13] both reported increased (-)-epicatechin within the blood following DC consumption; however both obtained DC which was manufactured to be precisely rich in flavanols by limiting the degradation, alkylation, and roasting of the cocoa seeds. The present study used Dove DC as Engler et al. [14] reported high associated polyphenol and (-)-epicatechin values; although, it was not stated whether the DC was specifically manufactured or was identical to the commercially available brand, as this may well contribute to a potentially lower flavanol and (-)-epicatechin concentration, thus may have potentially attenuated observed effects within this study. A caveat of the present study was that flavanol and NO were not directly measured thus the causation of the observed effects is speculated around flavanol and NO.

After 14 days consumption of DC, GET significantly increased by 11 % compared to WC and increased by 21 % compared to baseline. Similarly, $\overset{.}{V}{O}_2$ during moderate intensity cycling was consistently lower throughout the entire duration of cycling following consumption of DC (Fig. 2) compared to baseline and WC. However, $\overset{.}{V}{O}_2$ did not reach the threshold associated with statistical significance. The increases in GET complements findings by previous research using dietary nitrate supplementation which have been attributed to increased NO, which has been extensively shown to increase following regular consumption of DC [1, 2, 6].

Respiratory exchange ratio (RER) did not significantly differ between conditions (Fig. 4). These results conflict with Allgrove et al. [18], which reported lower RER values during exercise at 60 % $\overset{.}{V}{O}_{2 max}$, also indicating increased fat oxidation. Furthermore, consumption of DC was also reported to increase free fatty acid mobilisation during prolonged cycling following DC consumption, which could be viewed beneficial to those with goals tailored towards weight loss and athletes in need of conserving carbohydrate stores. However, the duration of the tests in this study were probably too short to elicit a decrease in RER. Moreover, DC does contain trace elements of caffeine and theobromine, which can independently increase free fatty acid mobilisation; although, the dose found is relatively small to make a substantial alteration in metabolism [25].

Unlike previously reported studies by Faridi et al. [26] and Sudarma et al. [13] systolic and diastolic BP was not seen to be significantly different between DC and WC. Systolic BP was attenuated by 6 % following the ingestion of DC to a healthier range (124 ± 7 mmHg), and diastolic BP increased by 6 % following ingestion of DC (74 ± 9 mmHg), which was also associated with healthier ranges. However, Faridi et al. [26] and Sudarma et al. [13] the participants in the present study were all healthy and had no irregular BP, possibly hindering the chance of finding a significant difference.

## Conclusion

This is the first study that has investigated the effects of DC consumption on GET, oxygen demands during a twenty-minute moderate cycle, $\overset{.}{V}{O}_{2 max}$ and performance of a two-minute maximal sprint. The primary outcome observed was DC consumption increased the work rate achieved at GET by 11 % compared to WC and 21 % compared to baseline (*P* < 0.05). Time trial distances following DC consumption were higher compared to baseline and WC (*P* < 0.05). Maximal oxygen consumption also increased following DC compared to baseline (*P* < 0.05), but was not significantly different to WC. However, despite oxygen consumption and RER being consistently lower throughout the twenty minutes of moderate intensity cycling, no significant difference was observed. Consequently, it can be concluded that ingestion of DC for 14 days reduced the oxygen cost of moderate intensity exercise and may be an effective ergogenic aid for short-duration moderate intensity exercise. However, future double-blinded studies will need to confirm this effect.

## References

[1] Bailey SJ, Wilkerson DP, DiMenna FJ, Jones AM (2009). Influence of repeated sprint training on pulmonary O2 uptake and muscle deoxygenation kinetics in humans. J Appl Physiol.

[2] Lansley KE, Winyard PG, Fulford J, Vanhatalo A, Bailey SJ, Blackwell JR (2011). Dietary nitrate supplementation reduces the O2 cost of walking and running: a placebo-controlled study. J Appl Physiol.

[3] Stamler JS, Meissner G (2001). Physiology of Nitric Oxide in Skeletal Muscle. Physiol Rev.

[4] Holt RR, Schramm DD, Keen CL, Lazarus SA, Schmitz HH (2002). Chocolate consumption and platelet function. JAMA.

[5] Engler MB, Engler MM (2004). The vasculoprotective effects of flavonoid-rich cocoa and chocolate. Nutr Res.

[6] Larsen FJ, Weitzberg E, Lundberg JO, Ekblom B (2007). Effects of dietary nitrate on oxygen cost during exercise. Acta Physiol.

[7] Corti R, Flammer AJ, Hollenberg NK, Luscher TF (2009). Cocoa and cardiovascular health. Circulation.

[8] Vlachopoulos C, Alexopoulos N, Stefanadis C (2006). Effect of dark chocolate on arterial function in healthy individuals: cocoa instead of ambrosia? Effect of dark chocolate on arterial function in healthy individuals. Curr Hypertens Rep.

[9] Kay CD, Kris-Etherton PM, West SG (2006). Effects of antioxidant-rich foods on vascular reactivity: review of the clinical evidence. Curr Atheroscler Rep.

[10] Steffen Y, Schewe T, Sies H (2007). (-)-Epicatechin elevates nitric oxide in endothelial cells via inhibition of NADPH oxidase. Biochem Biophys Res Commun.

[11] Schewe T, Steffen Y, Sies H (2008). How do dietary flavanols improve vascular function? A position paper. Arch Biochem Biophys.

[12] Fraga CG (2005). Cocoa diabetes and hypertension: Should we eat more chocolate?. Am J Clin Nutr.

[13] Sudarma V, Sukmaniah S, Siregar P (2011). Effect of Dark Chocolate on Nitric Oxide Serum Levels and Blood Pressure in Prehypertension Subjects. Acta Med Indones.

[14] Engler MB, Engler MM, Chen CY, Malloy MJ, Browne A, Chiu EY (2004). Flavonoid-Rich Dark Chocolate Improves Endothelial Function and Increases Plasma Epicatechin Concentrations in Healthy Adults. J Am Coll Nutr.

[15] Grassi D, Lippi C, Necozione S, Desideri G, Ferri C (2005). Short-term administration of dark chocolate is followed by a significant increase in insulin sensitivity and a decrease in blood pressure in healthy persons. AJCN.

[16] Taubert D, Roesen R, Lehmann C, Jung N, Schomig E (2007). Effects of low habitual cocoa intake on blood pressure and bioactive nitric oxide: a randomized controlled trial. JAMA.

[17] Berry NM, Davison K, Coates AM, Buckley JD, Howe PR (2010). Impact of cocoa flavanol consumption on blood pressure responsiveness to exercise. Br J Nutr.

[18] Allgrove J, Farrell E, Gleeson M, Williamson G, Cooper K (2011). Regular dark chocolate consumption's reduction of oxidative stress and increase of free-fatty-acid mobilization in response to prolonged cycling. IJSNEM.

[19] Wollgast J, Anklam E (2000). Review on polyphenols in Theobroma cacao: changes in composition during the manufacture of chocolate and methodology for identification and quantification. Food Res Int.

[20] Taubert D, Berkels R, Roesen R, Klaus W (2003). Chocolate and blood pressure in elderly individuals with isolated systolic hypertension. JAMA.

[21] Cooper KA, Donovan JL, Waterhouse AL, Williamson G (2008). Cocoa and health: a decade of research. Br J Nutr.

[22] Heiss C, Dejam A, Kleinbongard P, Schewe T, Sies H, Kelm M (2003). Vascular effects of cocoa rich in flavan-3-ols. JAMA.

[23] Schnorr O, Brossette T, Momma TY, Kleinbongard P, Keen CL, Schroeter H (2008). Cocoa flavanols lower vascular arginase activity in human endothelial cells in vitro and in erythrocytes in vivo. Arch Biochem Biophys.

[24] Fisher ND, Hughes M, Gerhard-Herman M, Hollenberg NK (2003). Flavanol-rich cocoa induces nitric-oxide-dependent vasodilation in healthy humans. J Hypertens.

[25] Costill DL, Dalsky GP, Fink WJ (1977). Effects of caffeine ingestion on metabolism and exercise performance. Med Sci Sport.

[26] Faridi Z, Njike VY, Dutta S, Ali A, Katz DL (2008). Acute dark chocolate and cocoa ingestion and endothelial function: A randomized controlled crossover trial. Am J Clin Nutr.

